# Peering Into the Past Century of Mountain Diversity Change by Uniting Two Modes of Remote Sensing

**DOI:** 10.1002/ece3.71507

**Published:** 2025-06-05

**Authors:** Julie A. Fortin, Jason T. Fisher, Eric S. Higgs

**Affiliations:** ^1^ School of Environmental Studies University of Victoria Victoria British Columbia Canada

**Keywords:** biodiversity, ecological change, landscape diversity, mountains, repeat photography, species distribution modeling

## Abstract

Mountain ecosystems are particularly susceptible to climate change and biodiversity loss as altitudinal diversity generates rare habitats and adapted specialist species, both sensitive to change. Mountain songbird diversity can be especially telling of land cover changes given breeding songbirds' strong patterns of habitat preference. However, most records of bird populations go back only a few decades, affecting baselines. Our aim was to examine changes in mountain diversity using a novel approach to analyze historical data that reaches nearly a century back in time. We repeated 46 historical survey photographs and used image analysis tools to quantify landscape change. In parallel, we generated species distribution models for 15 breeding songbird species in the study area. Based on the paired photographs, we modeled changes in bird occurrence. We then analyzed changes in Shannon diversity in terms of both land cover and bird occurrence. Forest cover increased over the past century at the expense of rarer alpine and riparian land covers, leading to decreased landscape diversity. This landscape homogenization resulted in declines in 5 species of songbirds (including 4 that breed in rare habitats), while 9 abundant forest‐breeding species were positively impacted, without substantial changes to the diversity of species in the community. We highlight shifts in species occurrence over a time interval not often captured by other methods. Historical photographs linked with species distribution modeling have potential for inferring global change for conservation and landscape management in mountain environments—some of the most challenging places to monitor.

## Graphical abstract

We investigate broad‐scale patterns in mountain diversity change over the past century. Our work shows a way to leverage nearly 100‐year‐old historical oblique photographs to peer farther into the past than is possible with decadal‐scale species monitoring datasets. We present our analysis for a single taxon, but our approach can easily be generalized to any number of taxa with occurrence data.

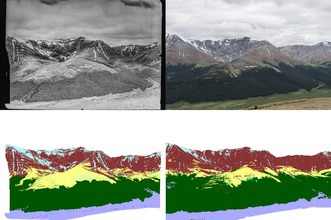

## 1 Introduction

Climate and land cover change are two of the dominant ecological challenges of our time, and are the main drivers of global biodiversity loss (Sala et al. 2000). Mountains in particular are regarded as sentinels of change because they have experienced higher‐than‐average rates of local warming (Pepin et al. 2022). The altitude gradients, complicated topography, and microclimates found in mountain ranges produce heterogeneous land cover, and the resulting variety of habitat types sustain a high diversity of both generalist and mountain‐adapted species (Ruggiero and Hawkins 2008). This heterogeneity can offer resilience (Graae et al. 2018; Lawrence et al. 2021; Oldfather et al. 2020), but it can also make mountain landscapes and species especially susceptible to pressures such as climate change, land use change, and anthropogenic disturbance (Hansen‐Bristow et al. 1988). Substantial changes in vegetation and species distributions have been documented in the world's mountain ranges, including in the North American western Cordillera (Inouye et al. 2000).

Bird species are diverse in alpine and montane ecosystems (Ruggiero and Hawkins 2008). Avian taxa make up an important part of the species and population declines observed in mountain systems worldwide because of their sensitivity to stressors such as habitat loss, forest encroachment, and wetland drying (Rittenhouse et al. 2012). Long‐term data are crucial to understanding biodiversity trends, especially given shifting baselines (Soga and Gaston 2018), yet data on bird diversity and abundance only exist a few decades into the past. One of the largest such datasets, the North American Breeding Bird Survey, was launched in 1966 and took decades to build in scope (Sauer et al. 2017). Thus, bird occurrence data contemporary with the significant landscape change occurring in North America throughout the 20th century, induced by climate change, rapid land development, and fire suppression, are sparse in temporal, spatial, or species coverage or completely absent (Boakes et al. 2010; Magurran et al. 2010).

In the absence of historical observations, it is possible to model species‐habitat associations with current data and use those models along with historical land cover data to “backcast” diversity and abundance before surveys became widespread (Hallman et al. 2021). The challenge emerging here, however, is that historical land cover data are sparse, too. The predominant sources of land cover information are remotely sensed aerial and satellite imagery, available at relatively high spatial and temporal resolutions only since the 1950s and 1970s, respectively.

Historical land cover information reaching earlier than both bird biodiversity data and remotely sensed imagery is available through oblique (i.e., land‐based) photographs. Beginning in the late 19th century, photography was used as a primary method for land surveys to create topographic maps in the mountainous regions of western Canada (Deville 1895). The resulting 120,000+ photographs have been remarkably well preserved in archives. The Mountain Legacy Project (MLP; mountainlegacy.ca) is an ongoing repeat photography project based on these historical collections. To date, the MLP has repeated over 10,000 historical images to document, describe, and study changes in mountain landscapes (Trant et al. 2015). The MLP has developed digital tools to quantify changes in paired photographs, such as the Image Analysis Toolkit that allows manual alignment of photographs and delineation, categorization, and quantification of pixel counts (Sanseverino et al. 2016). These images provide a lens into past landscapes and have been used to study ecological change in numerous ways (Fortin et al. 2018; Stockdale et al. 2019; Trant et al. 2020). The historical photographs in the MLP collection predate widespread bird surveys by several decades and were taken during a period of rapid anthropogenic land cover change in western North America as a consequence of colonial disruption and displacement of Indigenous peoples and practices.

**FIGURE 1:**
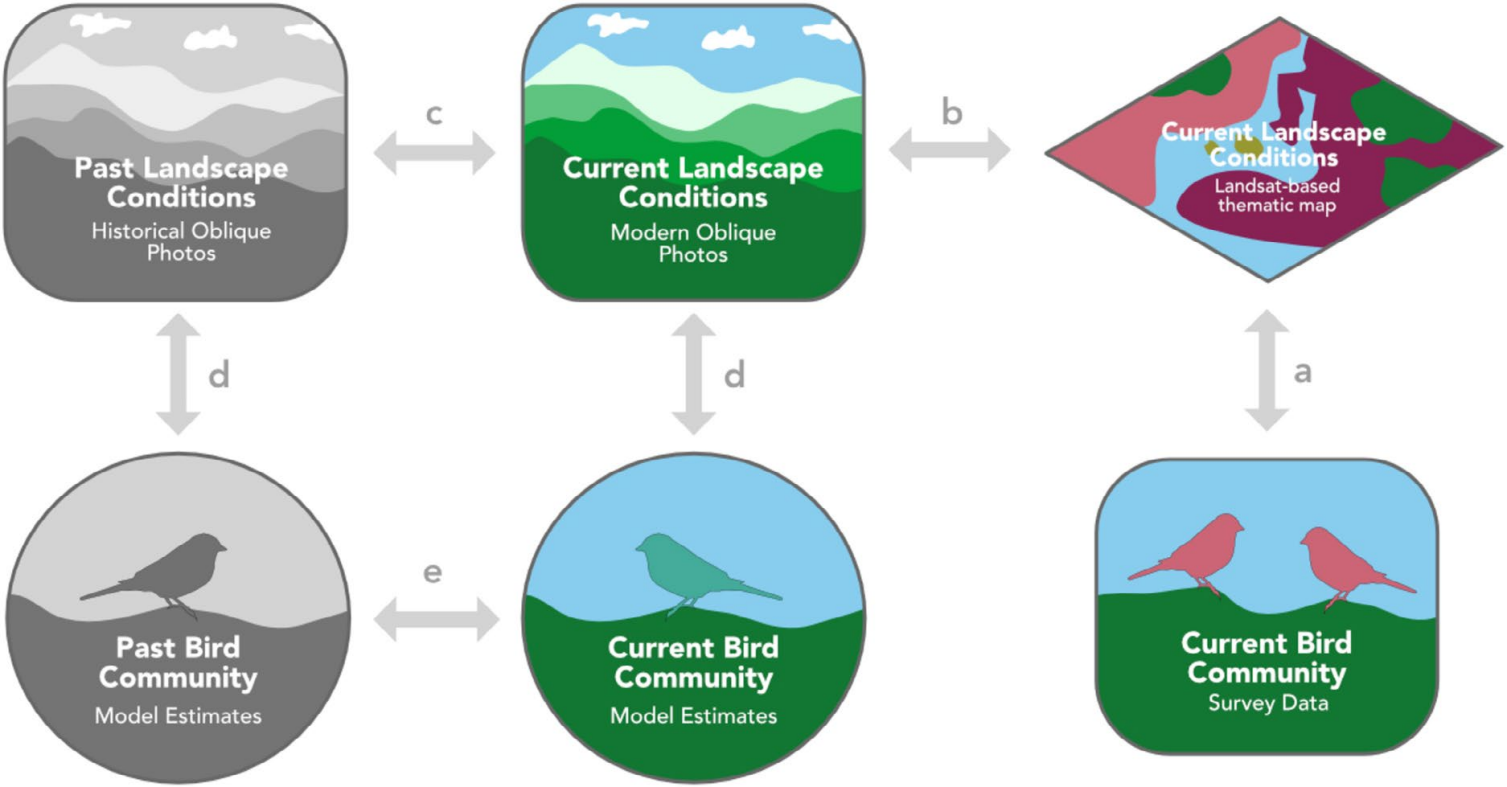
Conceptual model of approach: Creating and applying species distribution models to estimate bird diversity change. (a) Recent bird survey data was linked with recent landscape conditions to create species distribution models. (b) Land cover estimates from modern photographs were compared to land cover estimates from Landsat‐based thematic maps in Fortin et al. (2018). (c) Past and current landscape conditions were estimated from classifications of historical and modern photographs, respectively, and compared in Fortin et al. (2018). (d) Species distribution models generated in (a) were applied to past and current land cover composition estimated in (c) to estimate past and current occurrence of birds. (e) Bird community change was assessed by comparison of past and current estimates of bird occurrence probability.

We used this novel approach to quantify land cover change from historical and modern photographs from the MLP collection in a highly protected landscape in the Canadian Rocky Mountains. We selected this landscape because it is largely free from development and subject only to indirect anthropogenic impacts, such as climate change and suppression of wildfires and of Indigenous burning, to “stack the deck” against finding changes to bird communities—a highly conservative approach. We applied species distribution models to estimate the probability of occurrence of breeding songbirds at two time steps (Figure 1). We hypothesized that, given the observed homogenization of the landscape (Fortin et al. 2018), the avian community will have homogenized as well: with uphill treeline creep and valley bottom in‐filling, forest‐dwelling songbirds will have increased whereas species reliant on rarer herbaceous high‐elevation meadows and wet valley bottoms will have declined.

## 2 Methods

### 2.1 Study Area

**FIGURE 2:**
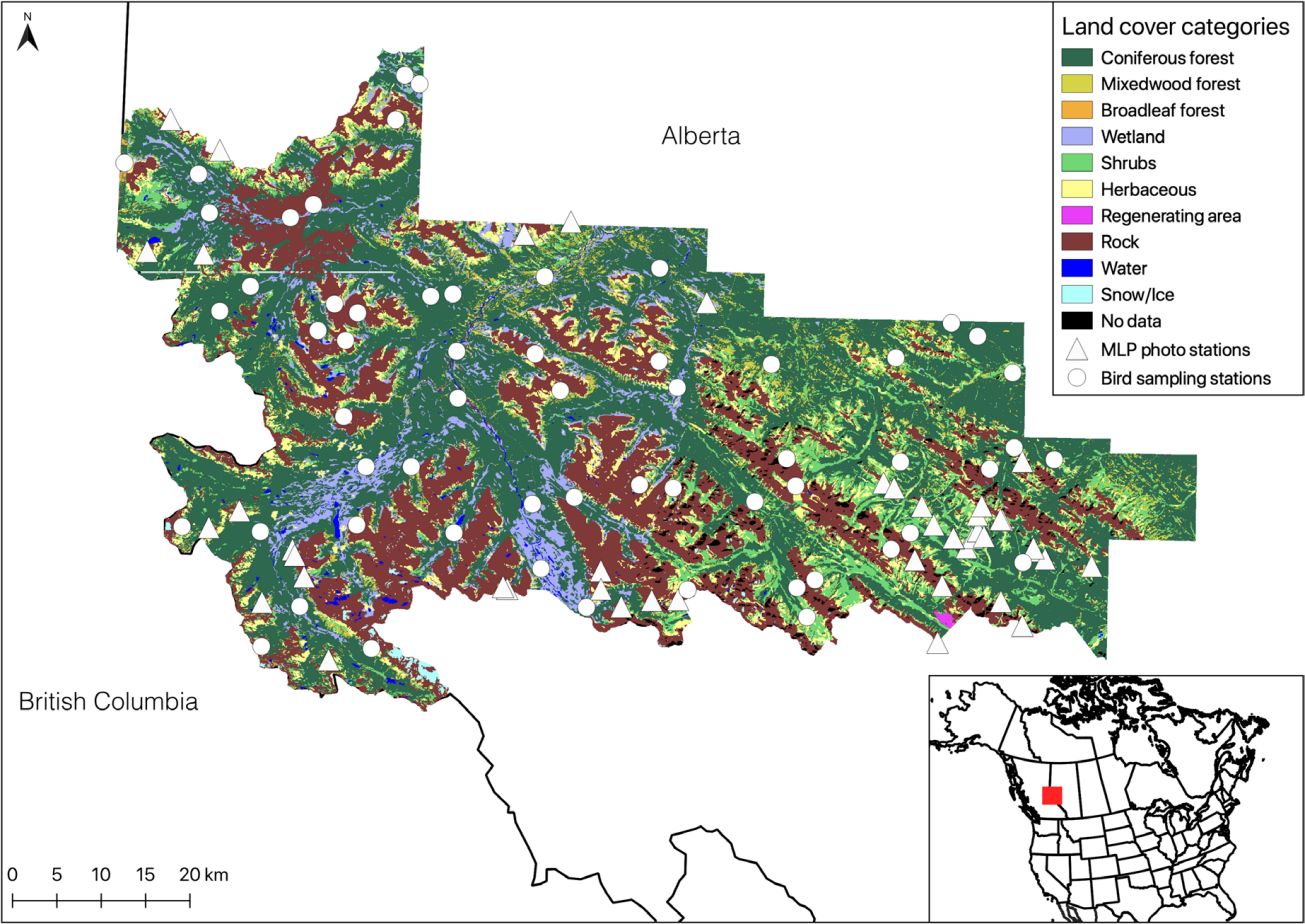
Study area map. Landsat‐based classified map (for the year 2010) of the Willmore Wilderness Park in west‐central Alberta, Canada, used to stratify bird sampling locations and to run species distribution models. Bird sampling stations are also shown, as are stations from which the historical and modern photographs were taken.

Our sampling frame is the front ranges of the North American Western Cordillera. Our sampling area is the Willmore Wilderness Park (WWP), a mountainous protected area in the Rocky Mountains of west‐central Alberta, Canada (Figure 2; Video 1). The WWP is protected by its own specialized legislation and hence is unique for its relatively light human use (Fisher et al. 2014; Stewart et al. 2016)—motorized vehicles are prohibited, so it is mainly accessed by hiking and horse packing trails—in stark contrast to other areas across the Rockies which are characterized by heavy recreational use and sometimes resource extraction. The topography of the WWP ranges from 300 m above sea level in the northeast to peaks as high as 3125 m on the western boundary. The WWP provides habitats ranging from jagged peaks to alpine meadows and shrubs to forests dominated by Engelmann spruce (*Picea engelmanni*) and subalpine fir ( *Abies lasiocarpa* ) to moist valley bottoms filled with black spruce ( *Picea mariana* ). The climate, topography, and vegetation shift from the west of the park toward the foothills of the east, with a greater diversity of deciduous trees in the east (Hall et al. 2000). The range of habitats in this area supports a diverse avian community: upwards of 60 songbird species have been detected in the park, including several species listed as “sensitive” according to Alberta's provincial ranking (Wild Species Status, 2015).

**VIDEO 1:** Overview of the study area: the Willmore Wilderness Park in Alberta, Canada. Video content can be viewed at https://onlinelibrary.wiley.com/doi/10.1002/ece3.71507

### 2.2 Land Cover Change

We quantified land cover change in 46 pairs of historical and repeat oblique photographs taken in the WWP nearly a century apart (Table S1). We selected images showing the full altitudinal range of available habitats (i.e., from peak to valley, eliminating photographs showing only alpine or barren land), covering the park's east–west vegetation gradient and facing all cardinal directions. Historical photographs were taken by federal or provincial surveyors between 1923 and 1953, and modern photographs were taken by the Mountain Legacy Project (www.mountainlegacy.ca) between 2007 and 2016 using repeat photography methods (Klett 2011; Webb 2010). While satellite imagery is more frequently used for studies of land cover, Fortin et al. (2018) found that oblique imagery provides a statistically comparable view at a landscape level in mountainous areas (where the assumption that satellite images are orthogonal to the land surface—and thus that pixels represent a constant area—does not hold).

We used a custom‐built tool to align the historical and repeat photographs with one another at a pixel level (Fortin 2018). We manually selected points corresponding to unchanging features on the landscape (e.g., mountain summits, corners of unmoved boulders) in both the historical and repeat photographs. Then the tool applied an affine transformation (translation, rotation and scaling while preserving straight lines) such that the selected point pairs are perfectly overlaid. The result is that the image pairs are nearly perfectly aligned (to within a few pixels, as was visually inspected using a “slider” feature) and thus we are confident that the images represent the same landscape and proportions of pixels will be comparable between the two images.

**FIGURE 3:**
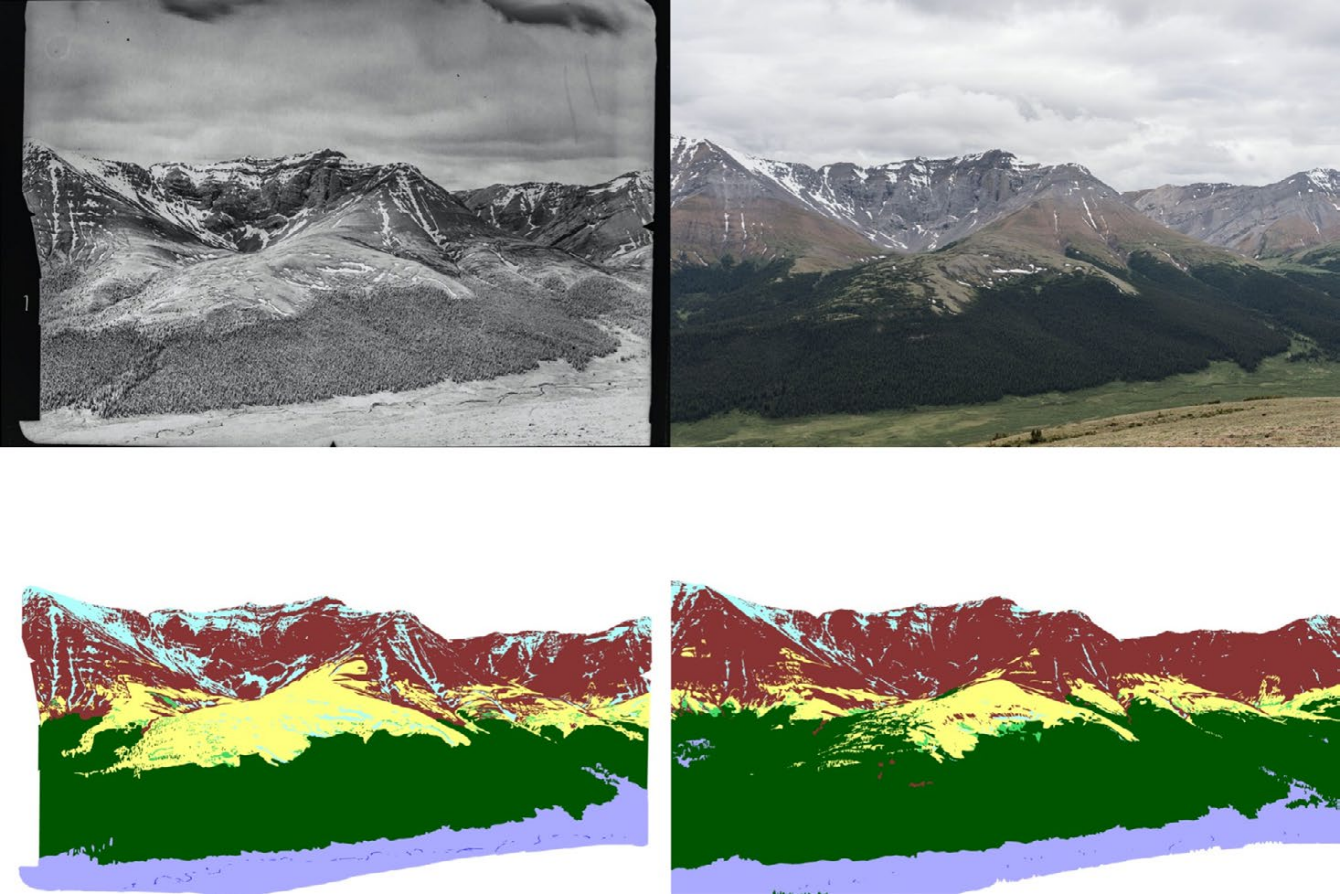
Example of photographs and land cover categorizations. Top left: Historical photograph taken by surveyor Michael Nidd in 1953. Top right: Repeat image taken by MLP in 2016. Bottom left and right: Classifications of the historical and repeat photographs, respectively. The legend is the same as in Figure 2.

We digitized the landscape in the photographs into 10 land cover categories: coniferous forest, mixedwood forest, broadleaf forest, wetland, shrubs, herbaceous, regenerating area, barren land, water, and ice/snow (Figure 3). Initial exploration of automated classification with the historical black‐and‐white photographs was poor due to limited spectral information. Therefore, despite it being more time‐intensive, we opted to manually delineate land cover in the photographs using a custom‐built application and a Wacom's Intuos PTZ‐930 pen tablet (Jean et al. 2015). Areas of high uncertainty (e.g., difficult to discern due to smoke or haze) were omitted from analysis, as was the foreground (any ground/rocks/elements that were within tens of meters from the camera), as recommended by Rhemtulla et al. (2002), due to disproportionately small areas covered by pixels in the foreground relative to the midground and background.

We used the MLP's Image Analysis Toolkit (Sanseverino et al. 2016) to count the proportion of pixels in each land cover category in the photograph pairs. We then compared the values for the historical and modern photographs to assess land cover change (Fortin et al. 2018).

In order to assess changes in landscape diversity, we calculated Shannon's diversity index for land cover types in each of the 46 pairs of photographs using the *vegan* package in R (Oksanen et al. 2019). We chose Shannon's index over other diversity indices for its sensitivity to rare land cover types (Nagendra 2002), which are of particular interest here. We ran Wilcoxon's signed‐rank tests using the *stats* package in R on the percent change of each land cover type and on the Shannon index to evaluate changes in landscape diversity (R Core Team 2015).

### 2.3 Bird Community Sampling

We chose to survey breeding songbirds because they exhibit particularly strong patterns of habitat preference and thus are good candidates for species distribution models (Chalfoun and Schmidt 2012). We sampled bird diversity in a mixed design, as in Fisher et al. (2014). Topographic and vegetation diversity is greatest along the east–west gradient, and a systematic grid of *n* = 30 points spaced 12‐km apart was designed to capture this diversity. However, because rare habitats are underrepresented in a systematic design, we also implemented a random stratified sampling design. We identified three land cover categories of interest: conifer, which is abundant (87% of the landscape according to satellite‐based classified maps); wetlands (6%), and mountaintop herbaceous meadows (7%), both of which are rarer. Wetlands included subalpine open and treed wetlands occurring in depressions and valley bottoms. Herbaceous regions predominantly occur between high‐altitude subalpine forests and rocky alpine patches. Ten sampling points were randomly assigned to each stratum in GIS, yielding an additional 30 candidate sites, for a total of 60. However, logistical constraints (poor weather) prevented us from sampling the full set of 60, so a subset of 43 sites was sampled. Half of all points were surveyed in May–June 2009, and the other half in May–June 2010, with sites randomly assigned to years.

At each site, songbird communities were sampled at 9 sampling units placed 300 m apart in a 3x3 square pattern with plots centered around each survey site (9 points including plot center). Birdsong was recorded via autonomous recording units (Shonfield and Bayne 2017) in the morning hours. We used digital recorders (Marantz, model PMD660) attached to radially directional acoustic microphones (Compression Zone Microphone, Riverforks Research Corp., Prince Albert, Saskatchewan, Canada). Audio recordings lasted 10 min per sampling unit, yielding a dataset of 90 min of bird calls per site, over 43 sites across the study area. Species were identified from recordings by professional bird transcribers using a double‐observer technique; we discretized data into the number of repeated detections of each species at each of the 9 sampling units per site as our response metric in generalized linear models (GLMs). Thus, the response metric ranged from 0 to 9, with each sampling unit within a site considered a Bernoulli trial wherein 0 = species not detected, and 1 = species detected.

### 2.4 Species Distribution Models

Land cover data was acquired by combining a province‐wide Landsat‐based thematic map with a digital elevation model to extract 16 potential habitat types (McDermid et al. 2009). From there, we combined categories distinguishing vegetation density by thresholds of crown cover to establish 10 simplified categories better suited for comparison with oblique photographs (i.e., “dense conifer”, “moderate conifer” and “open conifer” were merged to “conifer”). We checked for collinearity and used variance inflation estimation to confirm variables were sufficiently orthogonal (VIF < 3).

We used generalized linear models (binomial errors, log link) in R version 3.4.3 to relate the occurrence of breeding songbirds to land cover around each bird sampling site (R Core Team 2015; Zuur et al. 2009). The area around each site that should be quantified—the characteristic scale of selection (Holland et al. 2004)—varies among species and among landscapes. To identify the best‐supported model, we used a multi‐scale approach wherein we quantified land cover proportions within buffers around each site with radii ranging from 250 to 4500 m, in 250‐m increments. We used stepwise Akaike Information Criterion methods via the *MASS* package in R to identify the spatial scale and land cover categories that best explained species occurrence (Table 1) (Burnham and Anderson 2002; Venables and Ripley 2002). We used model diagnostics to assess model fits and retained only bird species with models that passed validation.

**TABLE 1:** Parameter estimates, with supporting evidence, for the variables included in the top model for each species.

Species	Best‐supported spatial scale (m radius)	Parameter	*β* estimate	Standard error	*t*‐value	*Pr* (>|*t*|)
Canada Jay ( *Perisoreus canadensis* )	1250	Intercept	−4.41	0.83	−5.29	7.2e‐06
		Coniferous forest	0.04	0.01	4.23	1.7e‐04
		Shrubs	0.06	0.03	2.54	0.02
Wilson's Warbler ( *Cardellina pusilla* )	1250	Intercept	−2.04	0.37	−5.51	3.8e‐06
		Herbaceous cover	−0.06	0.03	−1.82	0.08
		Shrubs	0.08	0.02	3.37	1.9e‐03
Savannah Sparrow ( *Passerculus sandwichensis* )	4250	Intercept	−5.26	0.81	−6.50	7.9e‐11
		Herbaceous cover	0.24	0.07	3.53	4.2e‐04
Golden‐Crowned Kinglet ( *Regulus satrapa* )	250	Intercept	−6.22	1.39	−4.46	8.1e‐06
		Coniferous forest	0.05	0.01	3.38	7.2e‐04
		Wetlands	0.05	0.01	3.31	9.3e‐04
		Shrubs	0.04	0.02	2.13	0.03
Ruby‐Crowned Kinglet ( *Regulus calendula* )	1500	Intercept	−4.85	1.22	−3.97	3.7e‐04
		Coniferous forest	0.03	0.01	2.34	0.03
		Broadleaf forest	0.73	0.36	2.03	0.05
		Wetlands	0.04	0.02	1.93	0.06
		Shrubs	0.05	0.03	1.81	0.08
Dark‐Eyed Junco ( *Junco hyemalis* )	1250	Intercept	−1.56	0.27	−5.75	1.7e‐06
		Herbaceous cover	−0.14	0.05	−3.11	3.7e‐03
		Shrubs	0.04	0.02	2.50	0.02
American Robin ( *Turdus migratorius* )	1500	Intercept	−0.80	0.37	−2.16	0.04
		Coniferous forest	−0.03	0.01	−2.98	0.01
		Broadleaf forest	0.49	0.26	1.90	0.07
Hermit Thrush ( *Catharus guttatus* )	500	Intercept	−2.36	0.53	−4.45	8.7e‐05
		Coniferous forest	0.01	0.01	2.00	0.05
		Broadleaf forest	0.94	0.33	2.84	0.01
		Wetlands	−0.02	0.02	−0.95	0.35
Pine Siskin ( *Spinus pinus* )	4500	Intercept	−1.02	0.40	−2.59	0.01
		Coniferous forest	0.02	0.01	2.19	0.03
		Broadleaf forest	−2.13	0.66	−3.25	2.6e‐03
American Pipit ( *Anthus rubescens* )	250	Intercept	0.21	0.20	1.09	0.28
		Coniferous forest	−0.08	0.02	−5.26	7.9e‐06
		Wetlands	−0.03	0.01	−2.57	0.01
		Broadleaf forest	0.73	0.23	3.13	3.5e‐03
Golden‐Crowned Sparrow ( *Zonotrichia atricapilla* )	250	Intercept	−0.11	0.22	−0.52	0.60
		Coniferous forest	−0.08	0.02	−4.24	1.6e‐04
		Wetlands	−0.03	0.01	−2.04	0.05
		Broadleaf forest	1.28	0.38	3.34	2.1e‐03
Swainson's Thrush ( *Catharus ustulatus* )	4000	Intercept	−9.52	2.00	−4.77	3.6e‐05
		Coniferous forest	0.07	0.02	3.87	4.8e‐04
		Wetlands	0.11	0.04	2.97	0.01
		Shrubs	0.14	0.05	2.92	0.01
		Broadleaf forest	1.47	0.66	2.22	0.03
Yellow‐Rumped Warbler (*Setophaga coronate*)	4500	Intercept	−1.01	0.93	−1.09	0.29
		Coniferous forest	0.03	0.01	2.52	0.02
		Herbaceous cover	−0.13	0.06	−1.98	0.06
Chipping Sparrow ( *Spizella passerina* )	250	Intercept	1.05	0.47	2.24	0.03
		Coniferous forest	−0.02	0.01	−2.97	0.01
		Wetlands	−0.02	0.01	−2.81	0.01
		Herbaceous cover	−0.01	0.01	−1.61	0.12
Varied Thrush ( *Ixoreus naevius* )	3750	Intercept	−3.79	0.89	−4.25	1.6e‐04
		Broadleaf forest	1.94	0.66	2.95	0.01
		Wetlands	−0.11	0.10	−1.12	0.27
		Shrubs	0.05	0.04	1.25	0.22

We employed a two‐stage approach to analysis. First, we used single‐season occupancy models (MacKenzie et al. 2018) to estimate songbird detectability (analysis by D. Mackenzie, Proteus Consulting, NZ). For each species, competing models assumed occupancy and detectability could vary by land cover. Model‐averaged estimates of p, and resulting estimates of probability of false absence (PFA), $\left(1-p\right)k$ where k is the number of sampling occasions, shows we detected birds reliably (Table S2). PFA was at worst 0.147, at best 0.001. While false absences are still possible, given very high detectability, we switched analyses to generalized linear models, as these are not as data‐hungry as occupancy models and sometimes have better ability to estimate habitat relationships (Fisher et al. 2020). We treated serial detection data—wherein identification of a bird by a transcriber is a detection of the species at that sampling unit, occurring in series through time and across repeated sampling units within a site—as true observations of 1's and 0's. In this manner 0's are interpreted as temporary emigration from the range of detection (a very likely interpretation) and a measure of site use, avoiding incorrectly partitioning of variance as error (Neilson et al. 2018; Stewart et al. 2018).

### 2.5 Bird Diversity Change

We back‐transformed the species distribution models from parameter estimates in log‐odds (Table 1) to the standard response scale (i.e., probabilities) according to a logit‐link function. We then used land cover proportion estimates from the historical and modern photographs to predict the past and current probability of occurrence of each bird species. For example, the top model for Canada jays ( *Perisoreus canadensis* ) on the standard response scale becomes

$$P=\frac{e^{\left(-4.42+0.04\times \mathrm{CF}+0.06\times \mathrm{SH}\right)}}{1+e^{\left(-4.42+0.04\times \mathrm{CF}+0.06\times \mathrm{SH}\right)}}$$

where P is the probability of occurrence of a Canada jay, CF is the percentage of coniferous forest in the landscape and SH is the percentage of shrub in the landscape, at a scale of 1250 m. We then ran Wilcoxon's signed‐rank tests on the past and current probabilities of occurrence for each species to test for changes over the last century.

**FIGURE 4:**
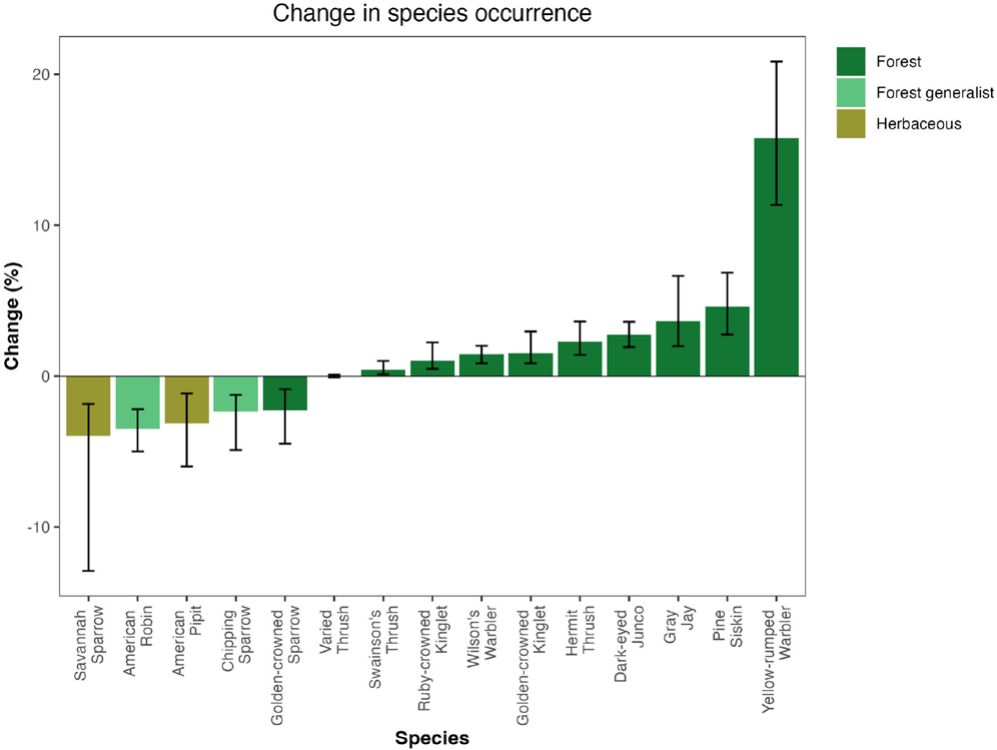
Change in probability of occurrence of bird species. Difference between contemporary and historical probability of occurrence, with 95% confidence intervals. Groupings come from the State of North America's Birds (2016) Report.

We linked each species to its major breeding habitat according to the State of North America's Birds report (State of North America's Birds 2016). We used these groupings to observe patterns in occurrence trends (Figure 4). We also compared our estimates to the long‐term percent change estimates from the Breeding Bird Survey for the Northern Rockies region which encompasses our study area in addition to the Rockies and Columbia mountain ranges within British Columbia and Alberta (Smith and Edwards 2021).

### 2.6 Sensitivity Analysis

In order to account for potential error propagation, we conducted a sensitivity analysis. The species distribution models we generated (Table 1) contained standard errors for each coefficient. We re‐ran our analysis with predictions generated using the lower and upper estimates of the models' *β* coefficients (i.e., *β* − SE, *β* + SE) (Figure S1).

## 3 Results

### 3.1 Land Cover Change

Coniferous forest cover increased mainly at the expense of wetlands and alpine meadows, decreasing landscape diversity over the 20th century. Coniferous forest cover increased from 40.1% (standard error 3.1%) of the landscapes surveyed to 52.5% (SE 3.7%); herbaceous cover declined from 15.5% (SE 2.2%) to 9.0% (SE 1.5%), as did wetland cover (4.8% SE 1.1% to 4.1% SE 0.9%). The remaining land cover categories did not change substantially in coverage between the historical and repeat photographs. The Shannon index of land cover diversity decreased from 1.2 (SE 0.04) to 1.0 (SE 0.04) on average. We provided additional detailed results on land cover changes observed in Fortin et al. (2018).

### 3.2 Species Distribution Models

We built species distribution models for 15 bird species, with a unique set of parameters and spatial scale that best explained occurrence for each (Table 1). Each species was either positively correlated with (more likely to occur in) or negatively correlated with (less likely to occur in) coniferous forest, broadleaf forest, shrub cover, herbaceous cover, and wetland; other land cover types did not have a strong influence on bird occurrence. For example, the probability of occurrence of hermit thrushes ( *Catharus guttatus* ) was positively correlated with coniferous and broadleaf forest, but negatively correlated with wetland, which is consistent with forest‐dwelling behavior known for this species.

About half of the species in our model set (8 of 15) had strongest associations with broadleaf forest cover as represented in the Landsat‐based classified map used to train the models. Yet broadleaf forest was a very small proportion of total land cover of our image samples, present in only 2 of 46 photograph pairs. This discrepancy in broadleaf detection is discussed in Fortin et al. (2018). Nevertheless, this does not invalidate our occurrence modeling results because other land covers that dominate our study region (coniferous forest, herbaceous and wetland cover) were also included in the models. The changes to these land covers were substantial enough to affect occurrence probability.

### 3.3 Backcasting Bird Diversity Change

Of the 15 bird species studied, nine increased in probability of occurrence, five declined, while one is not estimated to have changed substantially (Figure 4). Species that breed in coniferous forest tended to increase through time: Canada jay ( *Perisoreus canadensis* ), golden‐crowned kinglet ( *Regulus satrapa* ), ruby‐crowned kinglet ( *Regulus calendula* ), hermit thrush ( *Catharus guttatus* ), pine siskin ( *Spinus pinus* ), dark‐eyed junco ( *Junco hyemalis* ) and yellow‐rumped warbler ( *Setophaga coronata* ). Conversely, species that declined across the landscape preferred alpine herbaceous habitats for breeding: American robin ( *Turdus migratorius* ), American pipit ( *Anthus rubescens* ), chipping sparrow ( *Spizella passerina* ) and Savannah sparrow ( *Passerculus sandwichensis* ).

**FIGURE 5:**
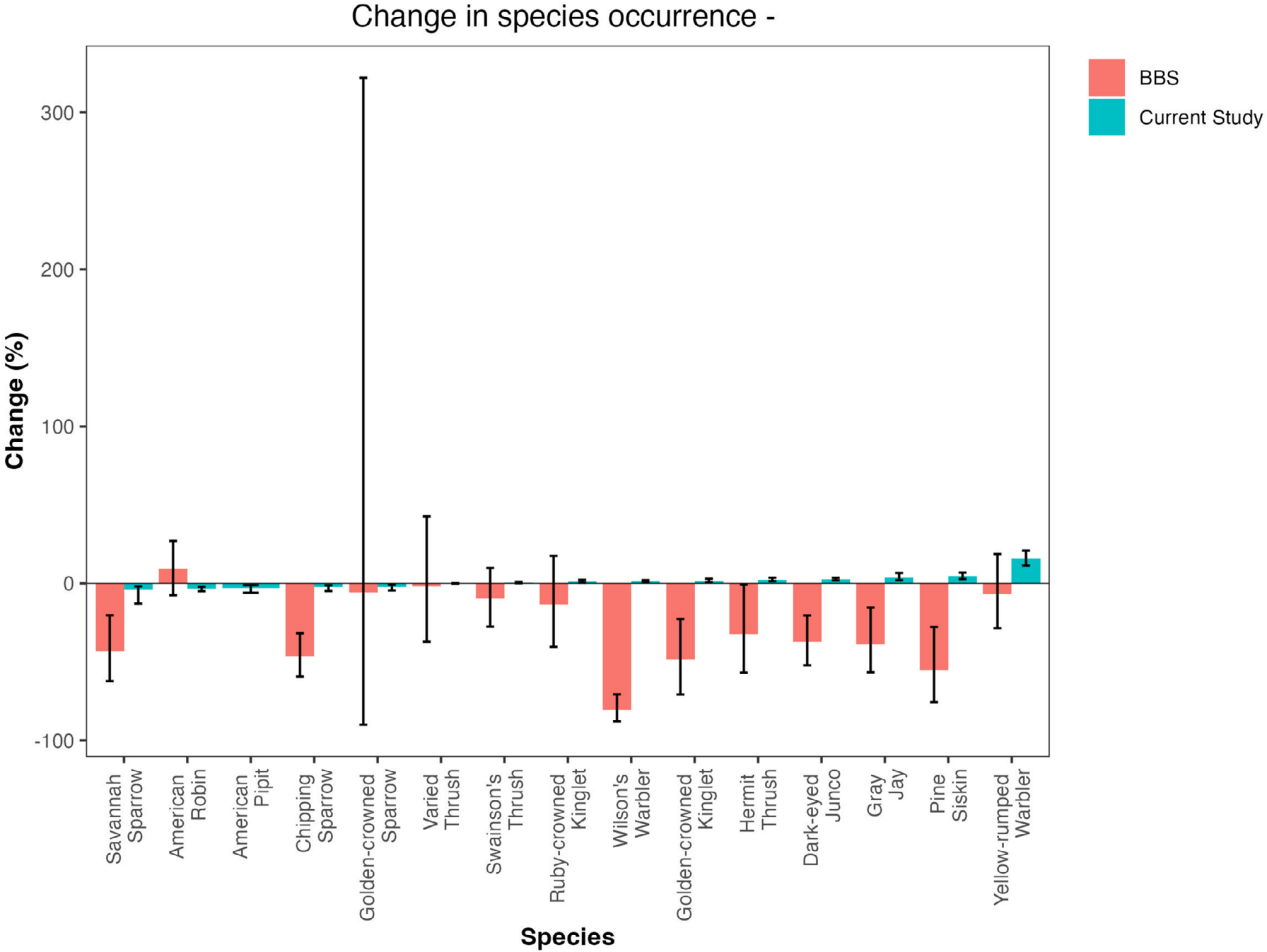
Change in probability of occurrence in each species in the current study (blue) versus long‐term trends from the Breeding Bird Survey (pink; Smith and Edwards 2021).

We compared our estimates to long‐term trends derived from the Northern Rockies Bird Conservation Region from the Breeding Bird Survey (BBS) (Smith and Edwards 2021) (Figure 5). We note agreement on the direction of change for 3 species, disagreement for 6 species, and overall much larger magnitudes of change in the BBS estimates.

We conducted a sensitivity analysis, re‐running our models using the lower and upper estimates of the models' *β* coefficients (i.e., *β* − SE, *β* + SE) (Figure S1). Our conclusions mostly did not change: for 13 species, the direction of projected change was the same through all scenarios.

## 4 Discussion

Marked changes in landscape diversity and in populations of several songbird species are estimated to have occurred in the 20th century, even in one of the most heavily protected areas of the Nearctic mountainous northwest. Forest encroachment and landscape homogenization have led to increases in forest‐breeding bird species and concomitant declines in species that preferentially breed in rarer alpine herbaceous and wetland habitats.

We chose this study area because of the minimal direct human impact. Indirect drivers of landscape change, however, have been shown to affect landscape‐scale vegetation in Rocky Mountain forests and are likely at play here. Long‐term processes such as climatic warming, shifts in precipitation patterns, and changes in wildfire regime from Indigenous burning to colonial fire suppression and exclusion (Keane et al. 2002) could explain the landscape changes observed. Other regions in the Rocky Mountains and other mountain systems across the globe are not only subject to the same indirect drivers but also experience direct landscape change through energy extraction, forest harvesting, recreation, and transportation. Thus, if we observed changes of this magnitude in such a protected landscape, we contend that large swaths of the western Cordillera and of mountains worldwide have likely experienced even greater shifts in avian communities.

Indeed, when comparing our results to long‐term trends derived from BBS across the Northern Rockies, we find that the BBS changes are often of much larger magnitude than our estimates, despite their shorter time frame (1970–2022). This could be due to the fact that land cover changes across the Northern Rockies have been much greater than in the highly protected area of the Willmore Wilderness Park, or perhaps differences in data collection methods.

The potential implications of landscape homogenization include altering biotic community structure (Rigal et al. 2022), as we observed; facilitating the spread of disturbances such as wildfire and invasive species (Risser 1984); and directly diminishing the value of mountains as hotspots of biodiversity. Notably, many rare bird species could not be modeled due to low detection rates, so all species assessed were labeled “secure” in recent provincial rankings—making our conclusions about change conservative. Despite their secure status, some species exhibited declines, again hinting at the importance of long‐term assessments and of extending temporal baselines to counter “shifting baseline syndrome” wherein past losses remain hidden from view, leading to spurious conservation assessment (Soga and Gaston 2018). Continued loss of rare habitat types such as alpine meadows and wetlands will further impact these populations.

There are assumptions and uncertainties underlying species distribution models that must be considered before using predictions to guide policy and conservation action (Wiens et al. 2009). First, our models did not include all factors that may affect species' occurrence; in particular, aspects such as landscape configuration and interspecific interactions were necessarily absent (Campbell et al. 2010; Fisher et al. 2013). Second, we assumed temporal stationarity: that each species' patterns of habitat preference did not change over the 20th century. Spatial non‐stationarity is an issue when extrapolating over large areas (Beresford et al. 2019; Osborne et al. 2007) but temporal stationarity is less known. We have assumed stationarity and contend that it is reasonable given the century timespan relative to eco‐evolutionary time scales. If bird species currently associated with rarer habitats were instead historically conifer‐associated, our backcasting would not hold. We could find no literature evidence nor logical reasons why this would be so. Third, our model specification (generalized linear model) requires both presence and absence data. Occupancy models showed we reliably detected birds when present, and so we treated serial detection data (i.e., detections of species by transcribers) as true observations in generalized linear models. Fourth, our model set was not representative of the entire avian community of the Rocky Mountains; only breeding songbird species that had sufficient observations in the auditory surveys to be modeled were examined. Sampling methods were stratified to ensure representation of rare habitat types (Fisher et al. 2014); nonetheless, sampling was skewed toward more common species. This may explain why, contrary to our hypothesis, we did not observe changes in bird diversity overall and implies that we are failing to detect losses or increases of rare species.

Mountain systems are seen as bastions of wilderness, but we show that even the most remote and protected have changed markedly in just a century. At a time when biological systems are changing at rapid rates, it is important to mitigate the effects of shifting baselines by framing current changes against historical data from an era that predates significant change. Leveraging older data sources can provide a lens into past ecological conditions. Historical photographs are one such example with broad geographic coverage; although they may not predate all land use change, they coincide with marked anthropogenic changes in the last century. Making use of this rich data source by combining methods from repeat photography, image analysis, and species distribution modeling allows for biodiversity change assessments with a baseline that extends farther back in time than possible with other sources. The magnitude of changes observed over approximately a century in this study is likely larger than would have been observed using data reaching only a few decades into the past.

There is significant potential to expand this work. By backcasting species occurrence using the methods we present here, systematic archival collections (e.g., the USGS Repeat Photography Project, rePhotoSA in South Africa, Alaska National Park Repeat Photography), smaller‐scale repeat photography projects (e.g., Madagascar—Kull 2005, Himalayas—Byers 2008), web‐scraped, crowd‐sourced, or citizen‐science images can be leveraged to study long‐term biodiversity changes. Recent advancements in land‐based image analysis such as georeferencing, viewshed mapping, and automated classification also show promise in scaling up this work. Future studies can increase the sample size, geographic coverage, and temporal range and could run models on other taxa for which species counts are available. This is particularly important in mountain environments, which are challenging places to monitor but have been especially susceptible to climate and landscape change.

## Author Contributions

**Julie A. Fortin:** conceptualization (equal), data curation (lead), formal analysis (lead), investigation (lead), methodology (lead), writing – original draft (lead), writing – review and editing (lead). **Jason T. Fisher:** conceptualization (supporting), data curation (supporting), methodology (supporting), supervision (equal), writing – original draft (supporting), writing – review and editing (supporting). **Eric S. Higgs:** conceptualization (equal), funding acquisition (lead), project administration (lead), supervision (equal), writing – original draft (supporting), writing – review and editing (supporting).

## Conflicts of Interest

The authors declare no conflicts of interest.

## Supporting information


Data S1.

## Data Availability

Code and data are already published and publicly available at DOI: 10.5281/zenodo.15353044. This includes the bird survey data and Landsat‐based classified map used to generate the species distribution models as well as the photographs, classifications, and model outputs used in theanalysis.
