# Supplementary material for: Peering Into the Past Century of Mountain Diversity Change by Uniting Two Modes of Remote Sensing
Source: Ecol Evol. 2025 Jun 5;15(6):e71507. doi: 10.1002/ece3.71507 (PMC12141755; doi:10.1002/ece3.71507)
Supplement: Supplementary file 1 — Data S1. [file ECE3-15-e71507-s001.docx]

# Supplementary Information

| **Surveyor** | **Year (historic)** | **Year (repeat)** | **Time spanned (years)** | **Station number** | **Image number (historic)** | **Cardinal direction** |
| --- | --- | --- | --- | --- | --- | --- |
| Lambart | 1927 | 2014 | 87 | 5 | 24 | East |
| Lambart | 1927 | 2011 | 84 | 15 | 2 | South |
| Lambart | 1927 | 2011 | 84 | 33 | 3 | West |
| Lambart | 1927 | 2007 | 80 | 35 | 261 | North |
| Lambart | 1927 | 2016 | 89 | 36 | 268 | North |
| Lambart | 1927 | 2007 | 80 | 37 | 280 | West |
| Lambart | 1927 | 2016 | 89 | 39 | 293 | East |
| Lambart | 1927 | 2007 | 80 | 42 | 320B | West |
| Lambart | 1927 | 2007 | 80 | 51 | 390 | East |
| Miller | 1928 | 2014 | 86 | 1 | 819 | East |
| Miller | 1928 | 2014 | 86 | 2 | 821 | West |
| Miller | 1928 | 2014 | 86 | 4.5 | 840 | West |
| Miller | 1928 | 2014 | 86 | 5 | 843 | East |
| Miller | 1928 | 2014 | 86 | 6 | 852 | South |
| Miller | 1928 | 2014 | 86 | 7 | 859 | South |
| Miller | 1928 | 2014 | 86 | 8 | 864 | West |
| Miller | 1928 | 2014 | 86 | 9 | 876 | East |
| Miller | 1928 | 2014 | 86 | 10 | 880 | South |
| Miller | 1928 | 2014 | 86 | 12 | 901 | West |
| Miller | 1928 | 2014 | 86 | 13 | 908 | South |
| Miller | 1928 | 2014 | 86 | 14 | 912 | North |
| Miller | 1928 | 2014 | 86 | 16 | 924 | South |
| Miller | 1928 | 2014 | 86 | 18 | 949 | East |
| Miller | 1928 | 2014 | 86 | 19 | 953 | East |
| Miller | 1928 | 2014 | 86 | 20 | 957 | East |
| Nidd | 1944 | 2012 | 68 | 11 | 1 | South |
| Nidd | 1944 | 2012 | 68 | 12 | 2 | South |
| Nidd | 1944 | 2012 | 68 | 15 | 3 | West |
| Nidd | 1944 | 2012 | 68 | 19 | 3 | East |
| Nidd | 1944 | 2012 | 68 | 21 | 2 | West |
| Nidd | 1946 | 2016 | 70 | 8 | 77 | South |
| Nidd | 1946 | 2016 | 70 | 12 | 121 | South |
| Nidd | 1947 | 2016 | 69 | 2 | 402 | South |
| Nidd | 1953 | 2016 | 63 | 3 | 500 | West |
| Nidd | 1953 | 2016 | 63 | 5 | 24 | South |
| Wheeler | 1923 | 2007 | 84 | 245 | 183 | North |
| Wheeler | 1923 | 2007 | 84 | 247 | 202 | North |
| Wheeler | 1923 | 2007 | 84 | 248 | 213 | North |
| Wheeler | 1923 | 2007 | 84 | 249 | 219 | West |
| Wheeler | 1923 | 2007 | 84 | 250 | 222 | West |
| Wheeler | 1923 | 2007 | 84 | 251 | 235 | North |
| Wheeler | 1923 | 2007 | 84 | 254 | 256 | East |
| Wheeler | 1924 | 2011 | 87 | 289 | 5 | East |
| Wheeler | 1924 | 2011 | 87 | 292 | 30 | West |
| Wheeler | 1924 | 2011 | 87 | 297 | 64 | West |
| Wheeler | 1924 | 2011 | 87 | 309 | 173 | West |

Supplementary Table 1. Metadata on the photographs used in the analysis. The Mountain Legacy Project (MLP) photographs can be identified based on the surveyor who took the historical photograph, the year of the survey, the station number and the image number. Images are freely available at explore.mountainlegacy.ca.

| Species | Total Detections | Naïve Occupancy | Model Averaged Occupancy | SE | Model averaged p | SE | Het Wght | Probability of false absence |
| --- | --- | --- | --- | --- | --- | --- | --- | --- |
| GCSP | 107 | 0.436 | 0.438 | 0.085 | 0.530 | 0.052 | 0.987 | 0.001 |
| AMPI | 102 | 0.487 | 0.497 | 0.082 | 0.515 | 0.044 | 1.000 | 0.001 |
| CHSP | 182 | 0.923 | 0.951 | 0.048 | 0.480 | 0.041 | 0.998 | 0.003 |
| YRWA | 175 | 0.821 | 0.863 | 0.069 | 0.477 | 0.031 | 1.000 | 0.003 |
| PISI | 106 | 0.795 | 0.913 | 0.109 | 0.325 | 0.048 | 0.534 | 0.029 |
| HETH | 81 | 0.539 | 0.654 | 0.143 | 0.305 | 0.053 | 0.984 | 0.038 |
| GCKI | 43 | 0.410 | 0.459 | 0.101 | 0.258 | 0.054 | 0.350 | 0.068 |
| SAVS | 42 | 0.282 | 0.349 | 0.098 | 0.231 | 0.043 | 1.000 | 0.095 |
| WIWA | 67 | 0.513 | 0.833 | 0.219 | 0.226 | 0.075 | 0.642 | 0.100 |
| RCKI | 43 | 0.487 | 0.587 | 0.108 | 0.208 | 0.037 | 0.723 | 0.122 |
| DEJU | 49 | 0.487 | 0.661 | 0.198 | 0.202 | 0.055 | 0.255 | 0.131 |
| AMRO | 73 | 0.641 | 0.995 | 0.010 | 0.199 | 0.028 | 0.985 | 0.135 |
| GRAJ | 73 | 0.539 | 1.000 | 0.000 | 0.192 | 0.020 | 1.000 | 0.147 |

Supplementary Table 2. Summary of assessment of detectability of species.

**
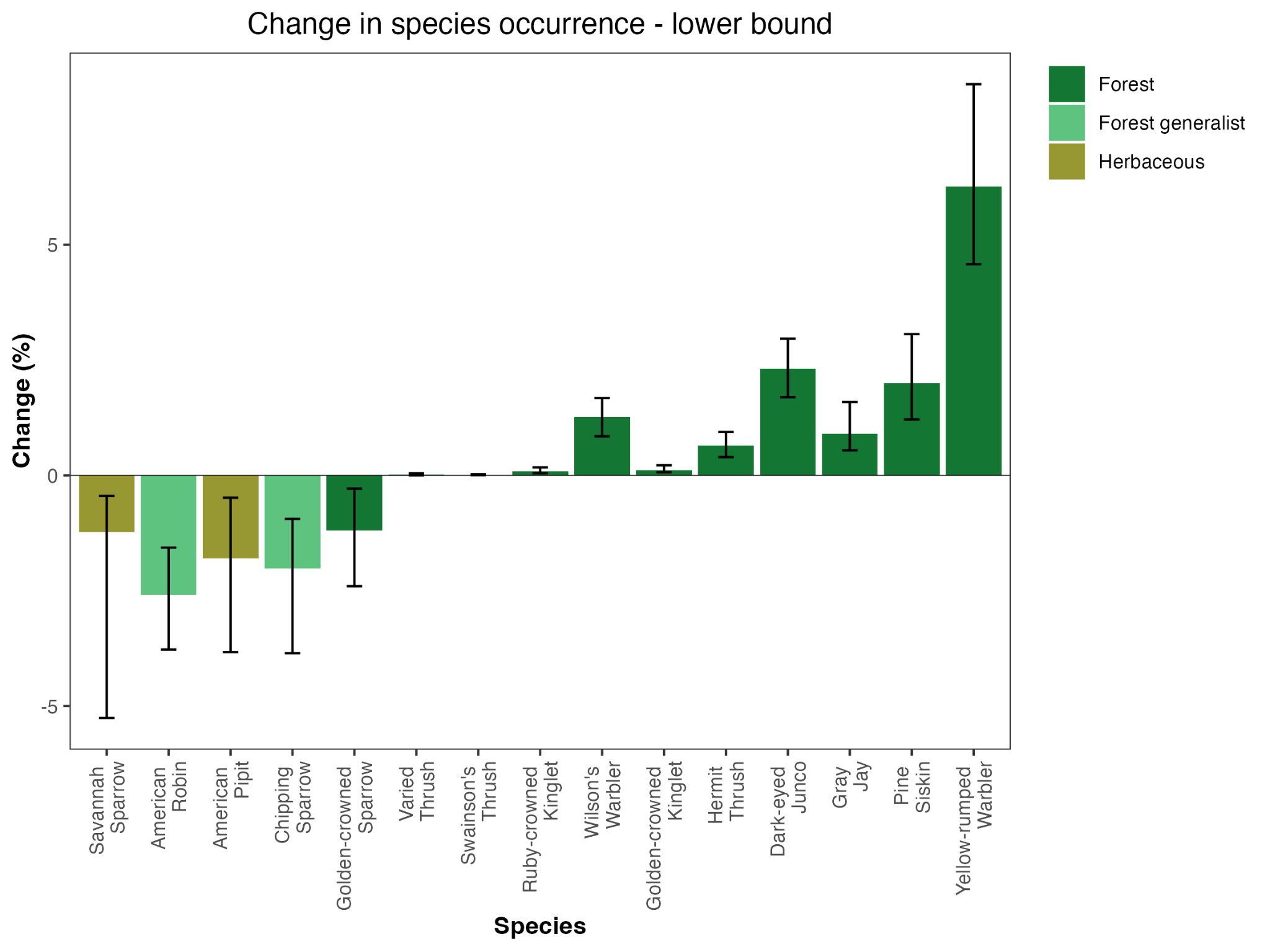

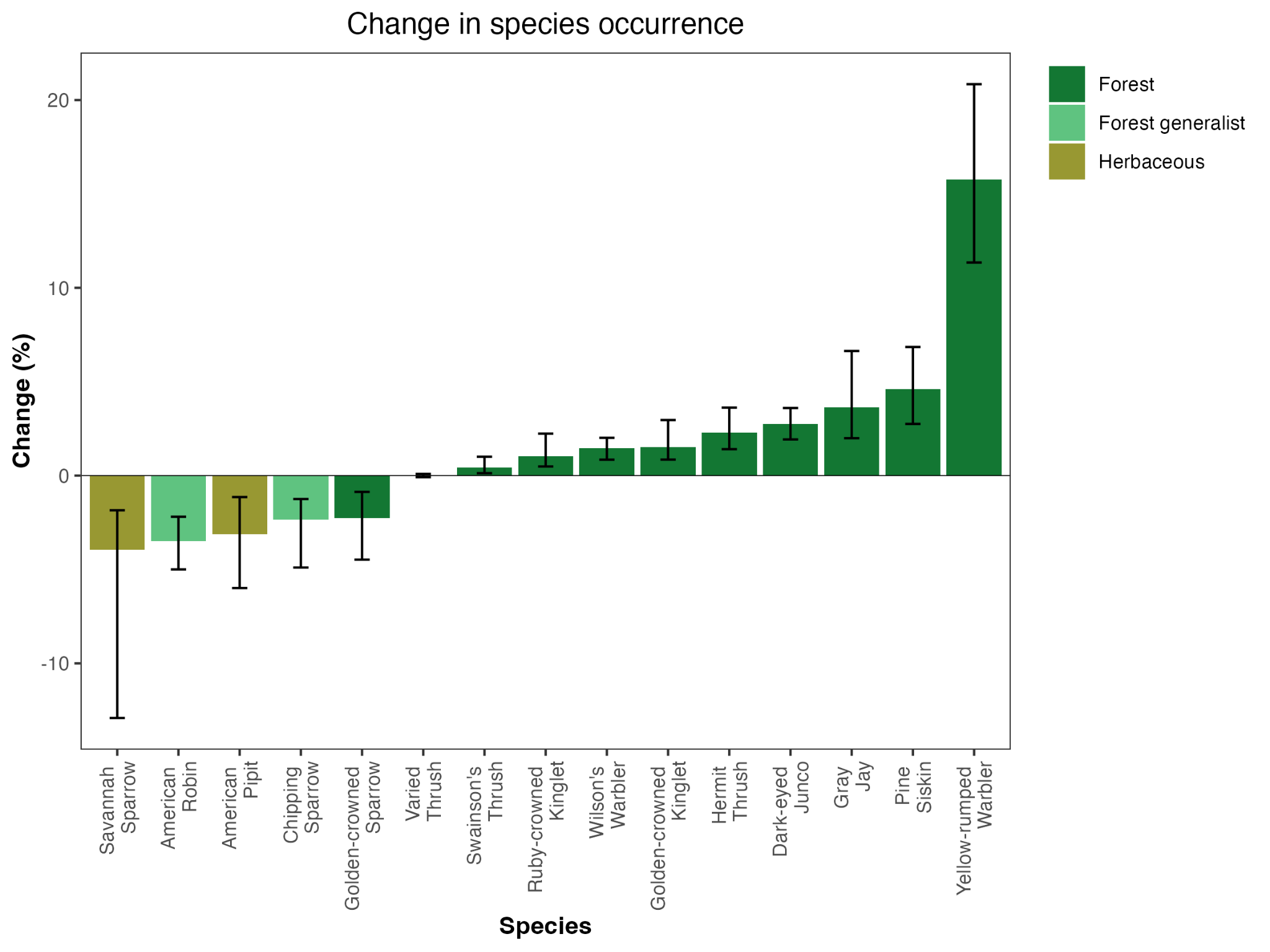

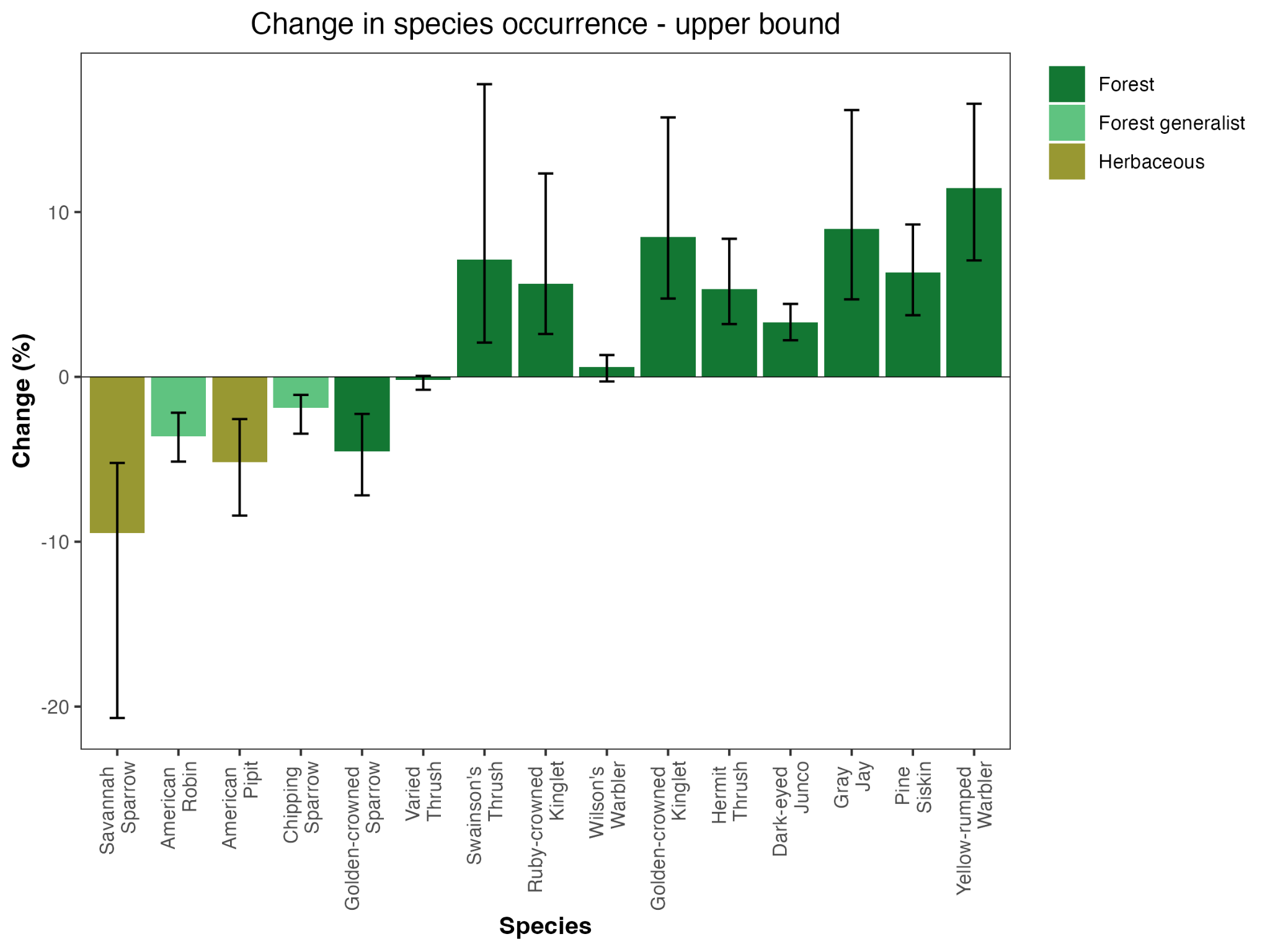
**

b

a

c

Supplementary Figure 1. Change in probability of occurrence of bird species using the species distribution model (a) β-SE, (b) β (same as Figure 4 of main text) and (c) β+SE to compute predictions.
